# Regulation of Microcystin-LR-Induced DNA Damage by miR-451a in HL7702 Cells

**DOI:** 10.3390/toxins11030164

**Published:** 2019-03-15

**Authors:** Lv Chen, Shu Yang, Cong Wen, Shuilin Zheng, Yue Yang, Xiangling Feng, Jihua Chen, Dan Luo, Ran Liu, Fei Yang

**Affiliations:** 1 Department of Occupational and Environmental Health, Xiangya School of Public Health, Central South University, Changsha 410078, China; chenlv@csu.edu.cn (L.C.); 18773152460@163.com (S.Y.); wencong941017@csu.edu.cn (C.W.); zhengshl@csu.edu.cn (S.Z.); yangy930806@gmail.com (Y.Y.); fengxl2011@csu.edu.cn (X.F.); chenjh@csu.edu.cn (J.C.); luodan_csu_2011@126.com (D.L.); 2 Key Laboratory of Environmental Medicine Engineering, Ministry of Education, School of Public Health Southeast University, Nanjing 210007, China; ranliu@seu.edu.cn; 3 Key laboratory of Hunan Province for Water Environment and Agriculture Product Safety, Central South University, Changsha 410083, China

**Keywords:** microcystin-LR, miR-451a, DNA damage, AKT1

## Abstract

Microcystin-LR is a cyclic heptapeptide hepatotoxin produced by harmful cyanobacteria. A panel of microRNAs containing miR-451a were found to be significantly changed in normal human liver cells HL7702 after exposure to microcystin-LR (MC-LR) in our previous study. However, the functions of miR-451a in hepatotoxicity induced by MC-LR remained unclear. The study aimed to investigate the impacts of miR-451a in HL7702 cells following treatment with 5 or 10 μM MC-LR. The comet assay indicated that MC-LR can influence Olive tail moment (OTM) in HL7702 cells. Furthermore, increase of miR-451a significantly repressed DNA damage and the protein expression level of γ-H2AX induced by MC-LR. Moreover, over-expression of miR-451a inhibited the expression level of p-AKT1 protein in cells following treatment by MC-LR. These results showed that miR-451a may protect from MC-LR-induced DNA damage by down-regulating the expression of p-AKT1, which provides new clues for the diagnosis and therapy policies for liver damage induced by MC-LR.

## 1. Introduction

Microcystins (MCs) are a series of cyclic heptapeptide toxins generated by cyanobacteria that pose serious threats to the ecological system and public health. More than 100 species of MCs isomers have been ensured, out of which microcystin-LR (MC-LR) is the most popularly studied and most hazardous [1]. To ensure the safety of drinking water for residents, the World Health Organization (WHO) suggested the concentration of MC-LR should not exceed 1 μg/L in drinking water [2,3].

Humans can absorb MC-LR by drinking water, aquatic products as well as recreational water and affect many organs including the liver, brain, heart, intestine, kidneys and reproductive organs [3]. It is reported that MC-LR is an effective liver cancer promoter and was divided into as a Group 2B carcinogen by the International Agency for Research on Cancer (IARC) [4]. Earlier studies have showed that MC-LR depressed the activity of protein phosphatase 1 and 2A and then induced cytoskeleton disruption, autophagy, apoptosis and DNA damage [5,6,7,8,9]. Some previous studies showed that MC-LR can induce DNA double strand breakage and oxidant-induced DNA damage in vivo and in vitro [10,11]. However, the specific signal pathway of DNA damage caused by MC-LR still remained undefined.

MicroRNAs (miRNAs) are a family of non-coding RNAs with 18 to 24 nucleotides. It can negatively regulate gene expression or suppress translation and stability of mRNA via pair combined with the 3’ untranslated regions of its target genes [12,13]. Nowadays, miRNA play a significant roles in numerous cellular processes, including differentiation, proliferation, apoptosis and metabolism and development of cancers [14]. Our previous studies have shown that some miRNAs were important in MC-LR-induced hepatotoxicity and miR-451a was most significantly down-regulated in the 10 µM MC-LR treated cells [15]. However, the mechanism of miR-451a in MC-LR-induced hepatotoxicity remains unclear.

The roles of miR-451a in the DNA damage and its potential function in MC-LR induced toxicity in hepatic cells were investigated in this study, which provides new clues for the diagnosis and treatment policies for liver damage induced by MC-LR.

## 2. Results and Discussion

### 2.1. MC-LR Exerts DNA Damage on HL7702 Cells

**Figure 1:**
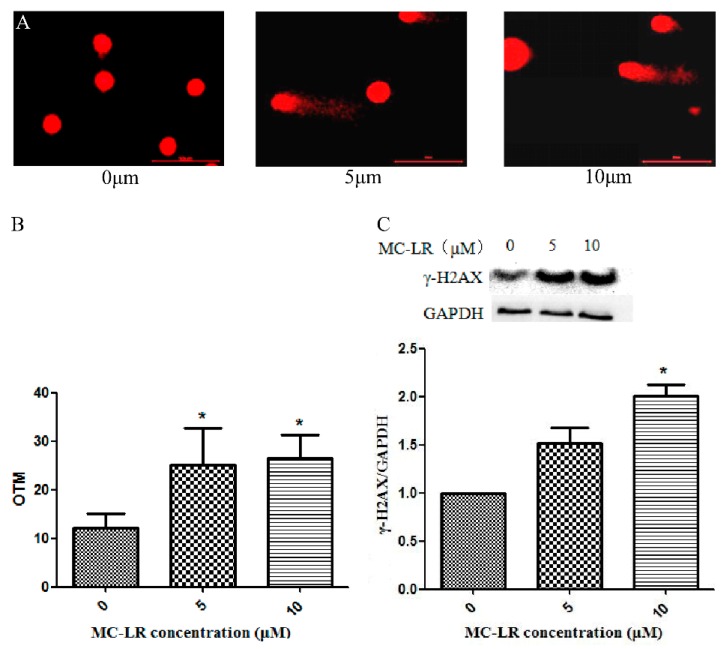
Microcystin-LR (MC-LR) induced DNA damage in HL7702 cells. HL7702 cells were exposed to MC-LR for 24 h. (**A**,**B**) DNA damage was detected by comet assay. (**C**) The protein expression of γ-H2AX was detected by Western blot. (* *p* < 0.05, experiments were performed in triplicate).

HL7702 cells were exposed to MC-LR (0, 5 and 10 μM) for 24 h and the DNA damage in HL7702 cells was determined with the comet assay and Western Blot. The data in Figure 1 represented the DNA damage in HL7702 cells of all experimental groups that was evaluated with Olive tail moment (OTM). DNA damage was significant increased after treatment with 5 and 10 μM MC-LR after 24 h compared with the 0 μM MC-LR group (Figure 1A). The mean value of OTM in 5 and 10 μM MC-LR treated cells was significantly increased in comparison to control group (Figure 1B).

γ-H2AX is a biomarker of DNA damage. Compared with the control group, the expression of γ-H2AX protein was significantly increased after a 24 h following 5 and 10 μM MC-LR and the level of γ-H2AX protein in the 10 μM MC-LR cells was statistically significant (Figure 1C).

Gaudin et al. found that DNA lesions were mainly induced in the liver, the kidney, the intestine and the colon after an intra-peritoneal injection. Furthermore, DNA damage rose after oral administration of MC-LR in mice blood cells [10], DNA damage were increased with dose and time dependent manner after exposing to MC-LR [11]. Thus, our results confirmed that MC-LR induces DNA damage and is similar to previous results.

### 2.2. Over-Expression of miR-451a Protects HL7702 Cells from MC-LR-Induced DNA Damage

**Figure 2:**
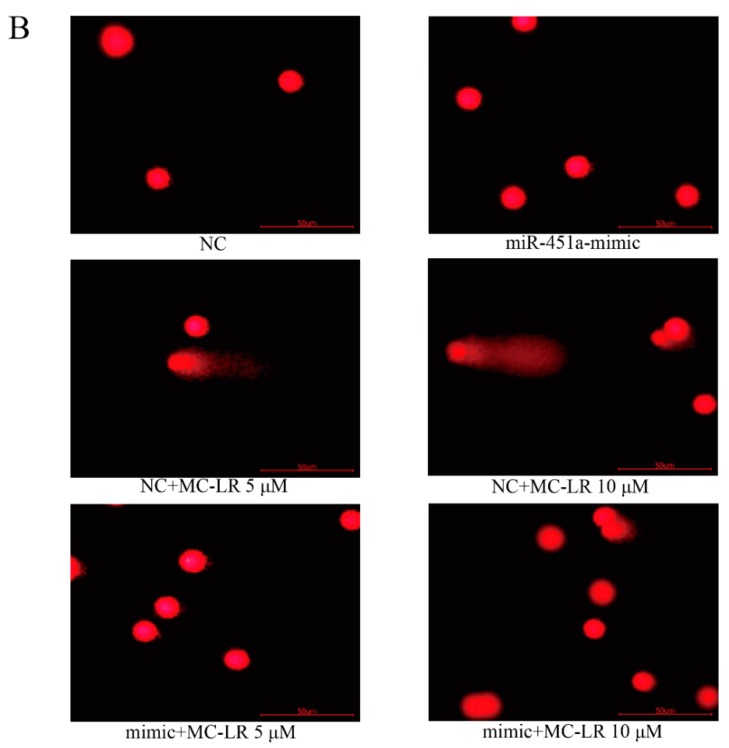

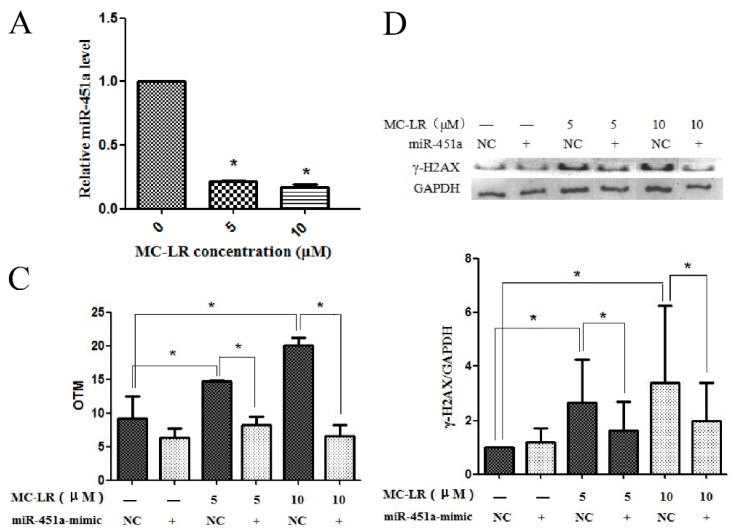
Effects of miR-451a on HL7702 cell DNA damage following exposure to MC-LR. (**A**) miR-451a expression was detected by qRT-PCR. HL7702 cells were transfected with miR-451a-mimic, negative control followed by treatment with MC-LR. (**B**,**C**) DNA damage was detected by comet assay. (**D**) The protein expression of γ-H2AX was detected by Western blot. (* *p* < 0.05, experiments were performed in triplicate).

Expression of many miRNAs including miR-451a, miR-4521 and miR-15b-3p substantially changed in HL7702 cells following exposure to MC-LR after 24 h in our previous study [15]. miR-451a was further studied in this study because miR-451a expression was significantly suppressed after exposure to MC-LR (Figure 2A). To investigate the potential function of miR-451a on cellular process, HL7702 cells were imported with miR-451a-mimic and their negative control followed exposure to 5 or 10 μM MC-LR for 24 h. DNA damage was decreased in cells imported with miR-451a-mimic compared to the negative control group (Figure 2B,C). High-expression of miR-451a inhibited the protein level of γ-H2AX in HL7702 cells in the presence of MC-LR (Figure 2D). These results confirmed that up-regulation of miR-451a suppressed the MC-LR-induced DNA damage. It has been reported that miR-451a is extensively dysregulated in many human malignancies including liver cancer [16,17], which suggests that miR-451a may play an important role in the tumorigenesis. A decrease in miR-451a expression was also observed in liver specimens of patients with non-alcoholic steatohepatitis [18]. Previous study has suggested differential expression of miR-451a in HL7702 cells induced by MC-LR, which indicated that miR-451a may be responsible for MC-LR-induced hepatotoxicity [15]. In this study, the expression of miR-451a was down-regulated after exposure of HL7702 cells to MC-LR and cellular DNA damage was displayed. After over-expression of miR-451a, MC-LR-induced DNA damage was significantly decreased, suggesting that MC-LR may induce DNA damage by regulating the expression of miR-451a.

### 2.3. The Expression of AKT1 and p-AKT1 Is Regulated by miR-451a

**Figure 3:**
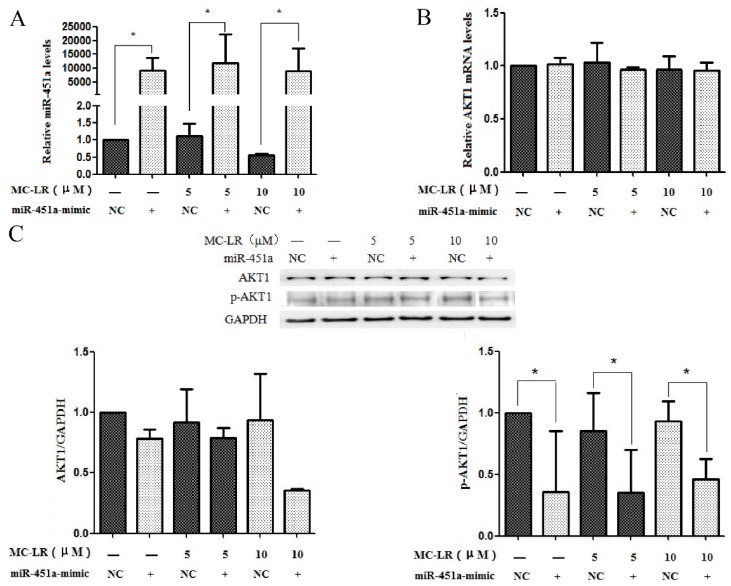
Protection of HL7702 cells from MC-LR-induced toxicity by miR-451a. HL7702 cells transfected with miR-451a-mimic, negative control were treated with MC-LR for 24 h (**A**,**B**). The mRNA levels of miR-451a (**A**) and AKT1 (**B**) were determined in HL7702 cells following transfection. (**C**) The protein levels of AKT1 and p-AKT1 were measured in HL7702 cells following transfection by Western blot. (* *p* < 0.05, experiments were performed in triplicate).

AKT1 may be a target mRNAs for miR-451a by literature search and mirTarBase database prediction. To make sure the effect of miR-451a on AKT1 regulation, the miR-451a expression was up-regulated in cells after transfected with miR-451a-mimic (Figure 3A). The mRNA and protein levels of AKT1 were not altered by the up-regulation of miR-451a (Figure 3B,C). However, the p-AKT1 protein levels were down-regulated in HL7702 cells after treatment with MC-LR (Figure 3C).

The miRNA degrades the target mRNA or inhibits translation by combining with 3’UTR of the target mRNA. AKT1 may be the target gene of miR-451a according to the prediction using the database (http://miRTarBase.mbc.nctu.edu.tw/). It has been confirmed that over-expression of miR-451a in papillary thyroid carcinoma significantly reduced the AKT1 and p-AKT1 protein levels [17]. AKT is a numerous of serine and threonine protein kinases that plays an important role in cell proliferation, cell migration and DNA damage. In addition, AKT is participated in the process of cellular metabolism, Protein formation and immune cell function [19]. Studies have shown that AKT can inhibit DNA damage repair [20,21,22,23,24]. AKT activation mediates MDM2 phosphorylation, resulting in the degradation of p53. The increase in p53 degradation results into the loss of DNA repair capacity, which ultimately leads to accumulation of DNA damage [20]. Our study found that miR-451a expression was significantly increased after transfection with miR-451a mimic but AKT1 mRNA and protein levels were not significantly altered, while p-AKT1 protein levels were significantly reduced. This indicates that AKT1 may not be the direct target gene of miR-451a. miR-451a may be participated in MC-LR-induced DNA damage by indirectly regulating phosphorylation of AKT1.

## 3. Conclusions

This study showed there was a vital linkage among MC-LR, miR-451a and AKT1 in HL7702 cells. The toxicity caused by MC-LR probably was mediated by inhibition of the miR-451a, which induced DNA damage indirectly regulating phosphorylation of AKT1. DNA damage induced by MC-LR in hepatocytes can be protected through increasing the intracellular expression of miR-451a. These results suggested new biomarkers for the prevention, diagnosis and treatment liver diseases caused by MC-LR.

## 4. Materials and Methods

### 4.1. Cell Culture and Treatment

HL7702 cell line was purchased from the Nanjing Huaao Biomedical Technology Co., Ltd. (Nanjing, China). Cells were cultured at 37 °C in a humidified 5% CO_2_ atmosphere containing RPMI-1640 medium (Gibco, Rockville, MD, USA), 10% (*v*/*v*) foetal bovine serum (FBS) (Gibco) and antibiotics (100 IU/mL penicillin, 100 mg/mL streptomycin (Sigma Chemical Company, St. Louis, MO, USA). Cells were subcultured every three days. Cells were grown with 5 × 10^4^ cells per well on 6-well plates and then MC-LR (Taiwan Algal Science Inc., Taiwan, China, purity ≥95%) was diluted into 0, 5 or 10 µM with RPMI-1640 medium and applied to the cells (experiments were performed in triplicated).

### 4.2. The Comet Assay

After exposure to MC-LR, 10 µL of cell suspension was mixed with 65 µL of 0.5% LMP agarose and added to fully frosted slides precoated with 1% NMP agarose. After solidification, the slides were lysed (2.5 M NaCl, 100 mM EDTA-Na_2_, 10 mM Tris, 1% sodium sarcosinate, 1% Triton X-100, 10% dimethyl sulfoxide, pH 10) 2 h at 4 °C. After the lysis, the lysed slides were immersed in the neutralizing solution (0.4 M Tris, pH 7.5) for 15 min. Then the slides were placed in the alkaline solution (300 mM NaOH, 1 mM EDTA-Na_2_, pH 13) for 20 min to allow DNA unwinding and subsequently electrophoresed for 30 min at 25 V, 300 mA. Finally, the slides were neutralized in 0.4 M Tris buffer (pH 7.5)-three times for 5 min, stained with propidium iodide (50 µg/mL) and photographed at 400× magnification using a fluorescence microscope. The comet pictures were analysed with CASP comet analysis software and the Olive tail moment of DNA was used to evaluate the level of DNA damage and a total of 50 randomly captured nuclei were examined from each slide in two independent experiments.

### 4.3. Cell Transfection

miR-mimics (control mimic and miR-451a-mimic) were purchased from Ribo Biological Company (Guangzhou, China). HL7702 cells were seeded in 6-well plates and allowed to reach 70% confluence after 24h. Lipofectamine™ 3000 (Invitrogen, Waltham, MA, USA) was used to transfect cells with DNA complexes according to manufacturer’s instruction and the cells were incubated at 37 °C for 24 h. Cells were grown with or without 5, 10 µM MC-LR for 24 h.

### 4.4. Quantitative RT-PCR (qRT-PCR)

Reverse transcription was conducted using the RevertAid First Strand cDNA Synthesis Kit (Thermo, Waltham, MA, USA) on S1000TM Thermal Cycler (Bio-Rad, Hercules, CA, USA). Quantitative PCR was performed using ChamQ SYBR qPCR Master Mix (Vazyme, NanJing, China) on a LightCycler96 Real-Time PCR System (Roche, Switzerland). All the procedures were repeated three times. The relative quantification values for each miRNA and mRNA were calculated by the 2^−∆∆Ct^ method using U6 and GAPDH as an internal reference, respectively. The sequences of mRNA primer pairs used were in Table 1.

### 4.5. Western Blot

After various treatments, total protein was extracted from the cells. Briefly, cells were lysed in an ice-cold extraction buffer (RIPA extraction buffer, 1 mM PMSF and 1× protease inhibitor cocktail). The concentration was monitored by the BCA method. Proteins were separated using sodium dodecyl sulphate-polyacrylamide gel electrophoresis (SDS-PAGE) and electroblotted onto a polyvinylidene fluoride (PVDF) membrane (Life Sciences, Waltham, MA, USA). Membranes were incubated with antibodies including primary rabbit anti-AKT1 (1:1000) (Abclonal, China), rabbit anti-phospho-AKT1 (1:1000) (Abclonal, China), mouse anti-phospho-γ-H2AX (1:1000) (Abcam, UK), mouse anti-GAPDH (1:1000) (Abclonal, Wuhan, China) at 4 °C overnight, followed by the addition of horseradish peroxidase-linked anti-mouse/rabbit IgG secondary antibodies (1:10,000) (Thermo) and ECL visualization of the bands.

### 4.6. Statistical Analysis

All calculations and statistical analyses were analysed by one-way analysis of variance (ANOVA), followed by the Dunnett’s *t* test with SPSS 13.0 (SPSS, Chicago, IL, USA). *p* < 0.05 is regarded as statistically significant.

## Figures and Tables

**Table 1:** qRT-PCR Primers in this study.

miRNA/mRNA	Forward Primer	Reverse Primer
miR-451a	5′-CGGCGAAACCGTTACCATTAC-3′	5′-GTCGTATCCAGTGCAGGGTCCGAGGT-3′
U6	5′-CTCGCTTCGGCAGCACATATACT-3′	5′-ACGCTTCACGAATTTGCGTGTC-3′
AKT1	5′-CCATCACACCACCTGACCAA-3′	5′-TCCCTCCAAGCTATCGTCCA-3′
GAPDH	5′-TCGGAGTCAACGGATTTGGT-3′	5′-TGGAATTTGCCATGGGTGGA-3′
